# Drug Resistance (Dapsone, Rifampicin, Ofloxacin) and Resistance-Related Gene Mutation Features in Leprosy Patients: A Systematic Review and Meta-Analysis

**DOI:** 10.3390/ijms232012443

**Published:** 2022-10-18

**Authors:** Xiang Li, Guoli Li, Jing Yang, Guangjie Jin, Yuting Shao, Yunhui Li, Pingmin Wei, Lianhua Zhang

**Affiliations:** 1 Department of Epidemiology and Health Statistics, School of Public Health, Southeast University, Nanjing 210009, China; 2 Department of Chronic Infectious Disease Control and Prevention, Jiangsu Provincial Center for Disease Control and Prevention, Nanjing 210009, China

**Keywords:** leprosy, drug resistance, genes mutations, dapsone, rifampicin, ofloxacin

## Abstract

Dapsone (DDS), Rifampicin (RIF) and Ofloxacin (OFL) are drugs recommended by the World Health Organization (WHO) for the treatment of leprosy. In the context of leprosy, resistance to these drugs occurs mainly due to mutations in the target genes (Folp1, RpoB and GyrA). It is important to monitor antimicrobial resistance in patients with leprosy. Therefore, we performed a meta-analysis of drug resistance in Mycobacterium leprae and the mutational profile of the target genes. In this paper, we limited the study period to May 2022 and searched PubMed, Web of Science (WOS), Scopus, and Embase databases for identified studies. Two independent reviewers extracted the study data. Mutation and drug-resistance rates were estimated in Stata 16.0. The results demonstrated that the drug-resistance rate was 10.18% (95% CI: 7.85–12.51). Subgroup analysis showed the highest resistance rate was in the Western Pacific region (17.05%, 95% CI:1.80 to 13.78), and it was higher after 2009 than before [(11.39%, 7.46–15.33) vs. 6.59% (3.66–9.53)]. We can conclude that the rate among new cases (7.25%, 95% CI: 4.65–9.84) was lower than the relapsed (14.26%, 95 CI%: 9.82–18.71). Mutation rates of Folp1, RpoB and GyrA were 4.40% (95% CI: 3.02–5.77), 3.66% (95% CI: 2.41–4.90) and 1.28% (95% CI: 0.87–1.71) respectively, while the rate for polygenes mutation was 1.73% (0.83–2.63). For further analysis, we used 368 drug-resistant strains as research subjects and found that codons (Ser, Pro, Ala) on RpoB, Folp1 and GyrA are the most common mutation sites in the determining region (DRDR). In addition, the most common substitution patterns of Folp1, RpoB, and GyrA are Pro→Leu, Ser→Leu, and Ala→Val. This study found that a higher proportion of patients has developed resistance to these drugs, and the rate has increased since 2009, which continue to pose a challenge to clinicians. In addition, the amino acid alterations in the sequence of the DRDR regions and the substitution patterns mentioned in the study also provide new ideas for clinical treatment options.

## 1. Introduction

Leprosy is a chronic infectious disease caused by *M. leprae* or *M. lepromatosis*, which may lead to irreversible damage to the skin, peripheral nerves, and even disability. The incubation period of the slow-growing viruses varies from 2 to 11 years, making treatment extremely difficult [1]. According to the WHO weekly report, there were 208,641 new cases around the world during 2018, and the transmission of leprosy continues in more than 100 countries, particularly in India (120,334 cases), Brazil (28,660 cases), and Indonesia (17,017 cases) [2,3].

Since 1940, dapsone (DDS) has been considered the most effective antibacterial and anti-inflammatory drug against *Mycobacterium leprae*. Nevertheless, the first DDS-resistant strain of *Mycobacterium leprae* was discovered in 1964 [4]. In 1981, WHO introduced multidrug therapy (MDT) for leprosy as a priority strategy to reduce drug resistance: the first-line drugs (rifampicin, ampicillin, and clofazimine) and second-line drugs (minocycline, ofloxacin, and clarithromycin). WHO also recommends a 1-year MDT course for multibacillary (MB) patients and a 6-month course for Paucibacillary (PB) [5]. Over time, new cases have been largely eliminated. In 1996, however, the first resistant strain to DDS, RIF, and OFL was identified. Relapse and recurrence cases remain a global public health problem associated with non-compliance with MDT or antimicrobial resistance (AMR), especially in MB patients [6,7,8]. As a first-line drug, there were few strains resistant to clofazimine. MmpS5-MmpL5 is the most important RND transporter protein in Mycobacterium tuberculosis, and its overexpression was associated with resistance to azoles (e.g., clofazimine). Nevertheless, MmpS5-MmpL5 is absent in Mycobacterium leprae, which partly explains the rarity of clofazimine-resistant strains [9].

**Figure 1:**
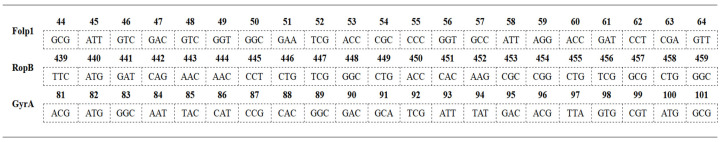
Resistance determining region (DRDR) sequences of target genes. FolP1, RpoB, and GyrA confer resistance to DDS, RIF and OFL, respectively, and the numbers indicate codon positions.

The failure of *Mycobacterium leprae* to grow in vitro hinders the investigation of AMR. There are two approaches available to detect drug resistance at present. The first one is to inoculate *Mycobacterium leprae* onto the foot pads of mice for culture and extract the tissues for testing [10]. In 1967, this method was used to detect drug resistance for the first time, such as DDS and fluoroquinolones. However, the growth of *Mycobacterium leprae* is slow and a long period of time (>1 year) is required to obtain the desired results, rendering the process both time-consuming and laborious. The second approach is to detect mutations by PCR-DNA sequencing. Studies have shown that mutations in the drug-resistance determining region (DRDR) in the Folp1, RpoB, and GyrA genes are responsible for resistance to DDS, RIF, and OFL [11,12]. The molecular-based detection method is more effective than the classic footpad experiment, reducing the turnaround time for diagnosis from months to hours. It has been shown that the DRDR region is situated between 44–64 loci of FolP1, 439–459 of RpoB, and 81–101 of GyrA (Figure 1) [13]. To date, many loci mutations associated with drug resistance have been identified. For instance, mutations within codon 53 (ACC→ATC) in the DRDR region of Folp1, ACC→ATC/GGC→GAC of RpoB, and mutations in codon 55/91 of GyrA confer drug resistance to Mycobacterium leprae [14,15,16].

There are many possible mechanisms of drug resistance in leprosy, such as changes in cell wall permeability and regulation of pump proteins. Genetic mutation detection remained the most recognized method with a high degree of sensitivity [17]. So far, although many papers have been published on the detection of drug resistance in *Mycobacterium leprae*, no publications have studied genetic mutations and amino acid substitutions in *this virus*. In this study, we investigated the global drug-resistance rate and gene mutation features of *Mycobacterium leprae* based on a meta-analytic approach. The paper aims to provide new ideas for the development of strategies to eliminate drug resistance and select more appropriate clinical treatment drug options.

## 2. Methods

### 2.1. Database and Search Strategy

The review was conducted in accordance with the established PRISMA protocol (Preferred Reporting Items for Systematic Reviews and Meta-Analyses) [18]. The review protocol has been registered in the International Prospective Register of Systematic Reviews (PROSPERO) (https://www.crd.york.ac.uk/prospero/display_record.php?ID=CRD42022340709, accessed on 2 October 2022). We limited the publication period from 1 January 2000 to 10 May 2022 and searched PubMed/MEDLINE, Web of Science (WOS), Scopus, and Embase databases for identified studies. Medical subject terms (MeSH) and free words were used as keywords for the literature search. In addition, the references in the identified papers were further screened for a relevant study. The main research keywords were “leprosy”, “Hansen’s Disease”, “resist”, “resistance”, “genes”, “mutations” and related terms. Finally, 1098 studies were identified from the database. The detailed database search strategy is shown in Supplementary File S1.

### 2.2. Study Selection

Duplicates were classified, annotated, and removed after exporting the retrieved papers to the reference software EndNote v.9.0 (Thomson Reuters, Stamford, CT, USA). Two researchers conducted the screening independently (Xiang Li, and Guoli Li), if disagreement still exists, a third author should be consulted. Finally, we evaluated the full text of the articles according to the inclusion and exclusion criteria.

### 2.3. Inclusion and Exclusion of Studies

Detailed inclusion and exclusion criteria were as follows. Inclusion criteria: (1) Drug resistance was detected by DNA sequencing recommended by WHO; (2) The studies that have reported mutations at target genes (Folp1, RpoB, and GyrA); (3) The studies should include accurate sample sizes (drug-resistant or none) and complete outcomes. (4) The included studies were primarily English-language reports (Full text). Exclusion criteria: (1) Diagnostic evaluation of experimental studies; (2) Animal-related studies; (3) Studies of low quality such as multiple publications, unpublished or unavailable full-text articles; (4) Reviews, conference reports, case reports, or social commentaries.

### 2.4. Data Extraction and Quality Assessment

Data were extracted independently by two investigators, and any disputes were resolved through discussion. The following information was extracted from the identified papers: region of the study, year of the study, sample type, study period, number of Mycobacterium leprae isolates, drug-resistance rates of Mycobacterium leprae isolates, gene mutation frequency, and locus mutation characteristics. The Joanna Briggs Institute recommended critical quality assessment inventory was used to assess the quality of all included studies (JBI scale) [18]. The results of the quality evaluation criteria are shown in the Supplementary File S2.

### 2.5. Statistical Analysis

Descriptive statistics were used to describe the overall search results and features of included studies with Microsoft Excel 2021. The extracted data were imported from Excel into Stata version 15.0. Forest plots evaluated the heterogeneity of the studies with the inverse variance (I^2^) statistic and Statistical quantities (χ^2^). Considering the differences among studies (I^2^ > 50%), the random effects model was applied at the 95% confidence level. Otherwise, a fixed effects model was used for I^2^ < 50%. Subgroup analysis was performed based on the study region, clinical treatment, drug-resistant strains, recurrence condition, study period, and sample size. In addition, we calculated the frequency of gene mutations and analyzed the probability of mutation at each locus with resistant strains. Finally, publication bias was assessed using funnel plots. The authors have provided the data extracted from the spreadsheet in the Supplementary File S3.

## 3. Results

### 3.1. Characteristics and Quality Assessment of Included Studies

**Figure 2:**
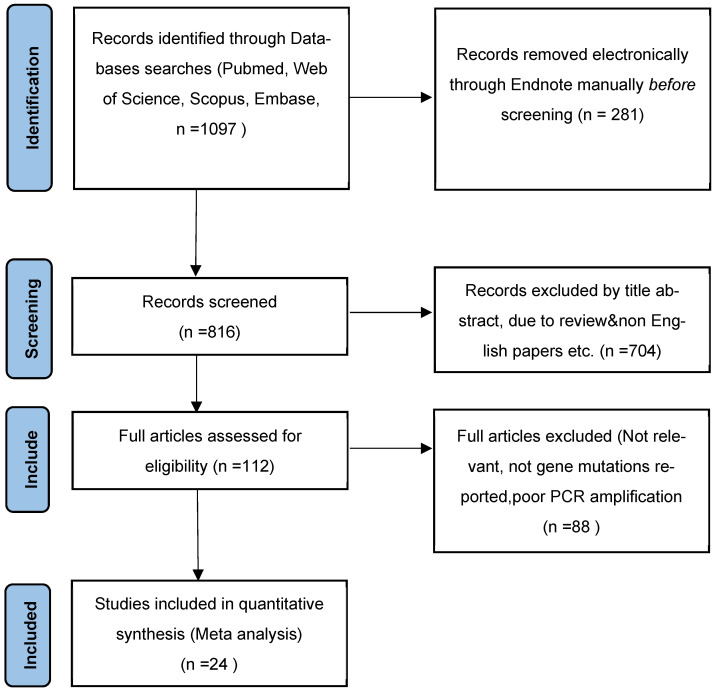
Flow chart of literature screening.

During the initial systematic search, 1098 studies were identified from the database. After eliminating duplicate papers by title filtering and excerpting, 113 articles remained. Articles with scores >5 were included based on predetermined criteria and the results of the JBI scale scores. Finally, there were 25 articles in total included in this meta-analysis after full-text evaluation. Figure 2 shows the search and selection process of the literature.

**Figure 3:**
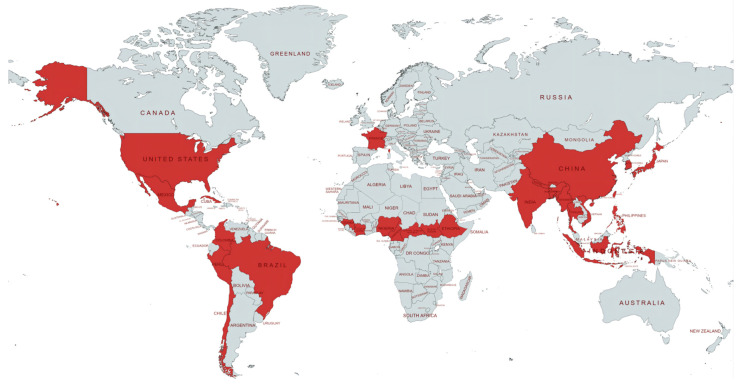
The distribution of countries covered in the 25 papers. Created with Mapchart (https://www.mapchart.net/, accession on 24 August 2022).

Table 1 and the Supplementary File S3 show the study characteristics and quality assessment. As a result, a total of 25 papers were included in this review, and there were 4349 leprosy patients included. Of these, 4128 were successfully amplified (94.92%), and 368 drug-resistant strains were obtained (RIF: 113, DDS: 145, OFL: 65, MDR: 57). A total of 6 papers were conducted before 2009, and 14 were conducted after 2009, while the remaining five papers were conducted outside of this temporal delineation criterion or without notification of the time of collection. A total of 20 (80%) papers were considered high quality because they provided accurate information on the number of drug-resistant patients among relapsed cases. Sixteen articles also provided accurate numbers of drug-resistant strains among MB patients (2394 cases) and PB patients (416 cases). Regarding regional distribution, 10 studies were from Southeast Asia, 8 from the Americas, 5 from the Western Pacific, and 1 from Europe (France) and Africa (Guinea). The study distribution map is shown in Figure 3. All articles reported the features of target gene mutations (Folp1, RpoB, GyrA), while 2 papers only reported RIF/RpoB.

### 3.2. Drug Resistance Analysis of Mycobacterium leprae

**Figure 4:**
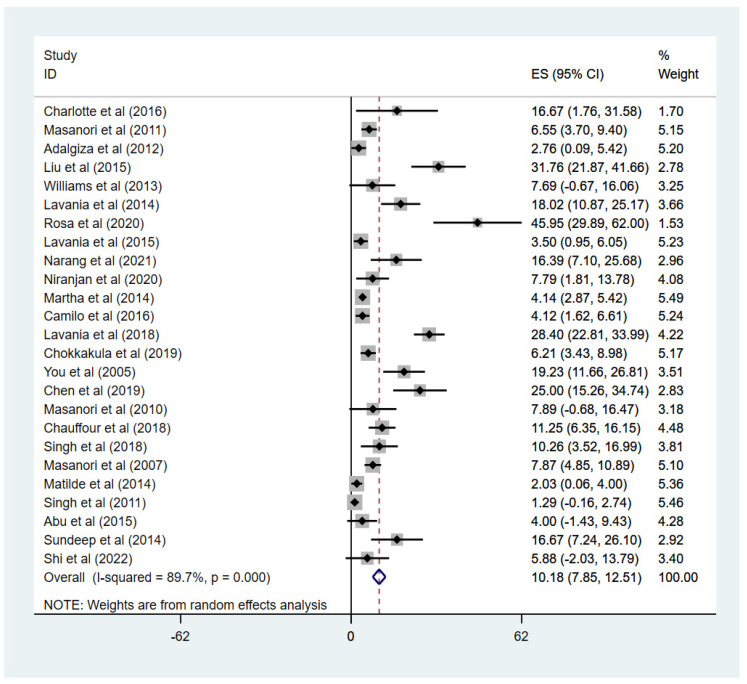
The forest plot of drug−resistance rates in Mycobacterium leprae. ES = drug−resistance rate, 95% CI = confidence interval. The incidence and 95% CI of the drug−resistance rate for each study are represented. Forest plot of drug−resistance rates of Mycobacterium leprae isolates in studies meeting inclusion criteria (n = 25) [14,19,20,21,22,23,24,25,26,27,28,29,30,31,32,33,34,35,36,37,38,39,40,41,42].

We calculated the drug-resistance rate of Mycobacterium leprae, and due to its heterogeneity I^2^ = 89.7% > 50%, a random effects model was adopted for analysis. As shown in Figure 4, the resistance rate of RIF, DDS, and OFL was 10.18% (95% CI: 7.85 to 12.51).

A subgroup analysis was performed due to the large heterogeneity in this study. The study areas were divided into six parts based on the WHO regional classification criteria. The results show that the highest resistance rate was found in the Western Pacific region (17.05%, 95% CI: 1.80–13.78). However, only 1 article in Europe and 1 in Africa was included, with a resistance rate of 11.25% (95% CI: 6.35–16.15) and 16.67% (95% CI: 1.76–31.58), respectively. We have also calculated the resistance rates between different drugs. The DDS rate was the highest (3.98%, 95% CI: 2.69–5.28), and the rate of MDR was 1.73% (95% CI: 0.83–2.63). After dividing patients into new and relapse groups, we found that the resistance rate was 7.25% (4.65–9.84) in new cases, which was lower than the rate of 14.26% (9.82–18.71) in relapsed patients. Temporal subgroups reported higher drug resistance after 2009 (11.39% vs. 6.59%). In addition, studies with <100 isolates had higher resistance rates than those with more than 100 isolates. Details of the results are shown in Table 2.

### 3.3. Mutation Analysis of Drug Resistance Genes in Mycobacterium leprae

In this review, 20, 22, and 16 articles studied Folp1, RpoB, and GyrA genes 139, 129, and 83 times. The results showed that the mutation rates of Folp1, RpoB and GyrA were 4.40% (95% CI: 3.02–5.77), 3.66% (95% CI: 2.41–4.90) and 1.28% (95% CI: 0.87–1.71), respectively, as it was shown in Table 3.

Based on the 382 drug-resistant strains, it can be seen that the RpoB gene involved the largest variety of mutations and the GyrA the least, as it was shown in Table 4. In the DRDR region of RpoB, the mutation rate in the codon encoding Ser was 54.17% (95% CI: 53.02–69.40), indicating that Ser had the highest probability of mutation. The codon encoding Pro has the highest rate of mutation (83.97%, 95% CI: 77.58 to 90.37) in Folp1. The most common mutation loci in GyrA was Alanine (Ala), with a probability of 75.18% (95% CI: 68.54 to 88.62). In addition, there were insufficient data from the studies to analyze rare mutations, such as Arg in RpoB, Ala in Folp1, and Leu in GyrA.

### 3.4. Subgroup Analysis of Site Mutation Patterns at Folp1, RpoB, and GyrA

**Figure 5:**
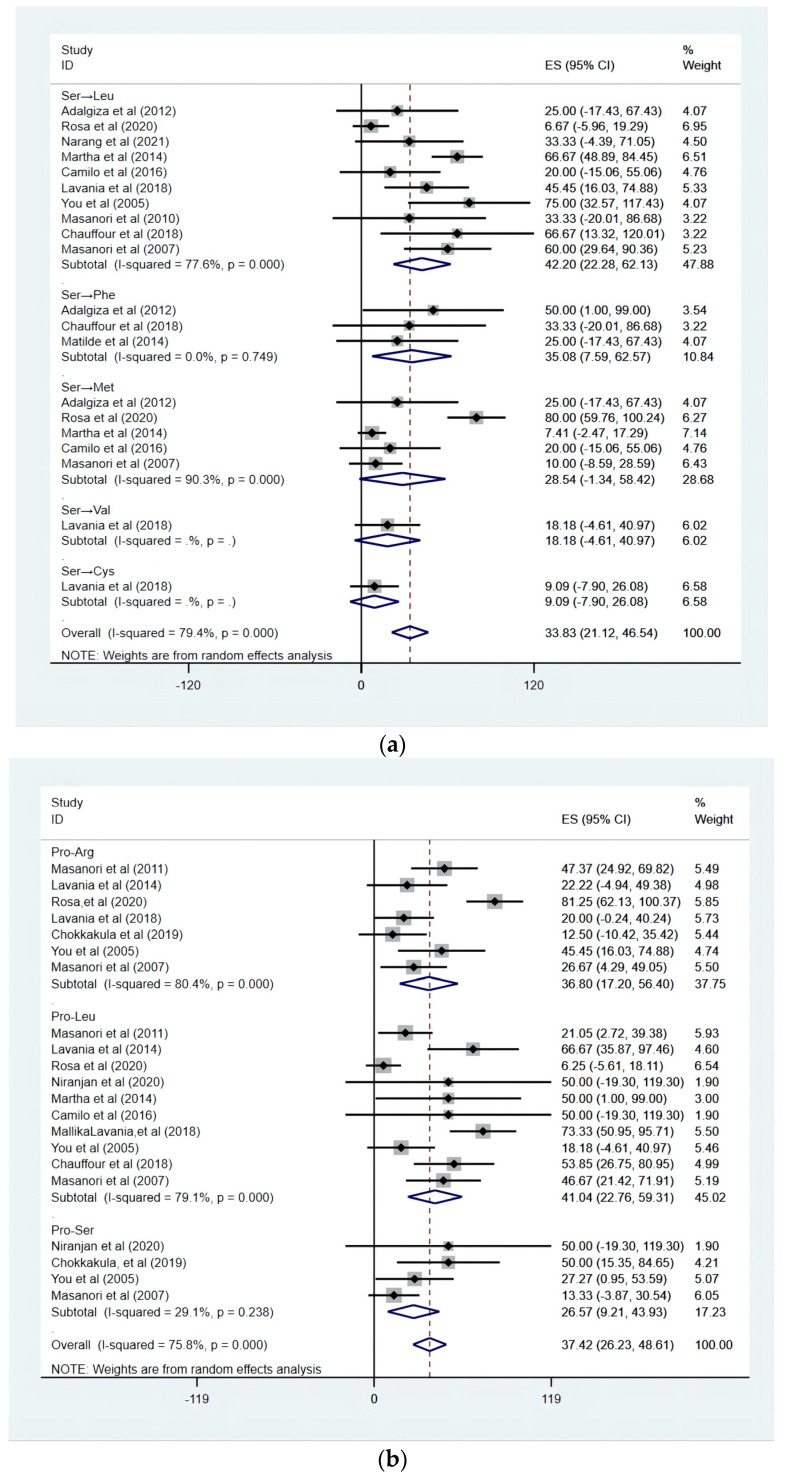
The Ser, Pro mutation forest plot. (**a**) The forest plot of the Ser mutation in RpoB. (**b**) The forest plot of the Pro mutation in Folp1 [14,20,21,24,25,27,28,29,30,31,32,34,35,37,38].

A random effects model was used to conduct a subgroup analysis of the codon with the highest mutation rate. As the result indicated, the most common mutation pattern in Folp1 was Pro→Leu, followed by Pro→Arg, with an occurrence of 41.04% (95% CI: 22.76–59.31) and 36.80% (95% CI: 22.76–59.31). On the DRDR region of RpoB, Ser→Leu was the most common mutation pattern in Folp1 (42.95%, 95% CI: 22.65–63.25), followed by Ser→Phe (ES: 35.08%, 95% CI: 7.59–62.57). We did not perform a subgroup analysis for Ala, since a majority of the included publications were Ala→Val (92.65%, 63/68), as shown in Figure 5.

### 3.5. Publication Bias Analysis

**Figure 6:**
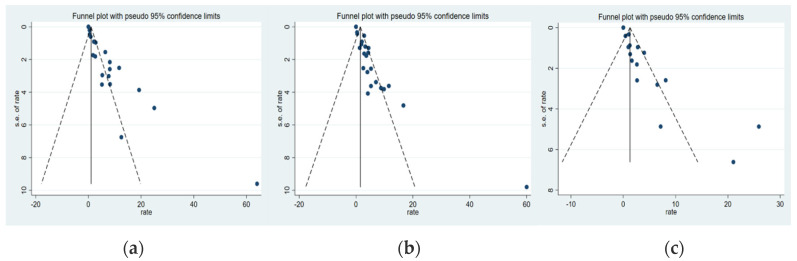
Funnel plot of gene mutation in Mycobacterium leprae. (**a**) Funnel plot of Folp1; (**b**) Funnel plot of RpoB; (**c**) Funnel plot of Folp1.

The funnel plots were used to assess publication bias, and the standard error was plotted against the gene mutations. The results showed a symmetrical distribution, indicating the absence of publication bias, as it was shown in Figure 6. The main causes of bias are the following. First, leprosy has been completely eradicated in some areas, with an insufficient number of papers available for research. Second, positive results may be more easily published. In order to avoid publication bias and heterogeneity, we attempted to reduce their impact on the analysis through random effects models and subgroup analysis but were still unable to eliminate the impact on the interpretation of the pooled results.

## 4. Discussion

Intensive chemical treatment has led to a significant reduction in leprosy patients. However, the emergence of multidrug-resistance (MDR) remains a major public health problem in leprosy control. Thus, we conducted a meta-analysis to describe the drug resistance (DDS, RIF, OFL) and gene mutation features of leprosy, aiming to provide recommendations for alternative treatment options.

After scoring by the JBI scale, 25 articles were included (score ≥ 5). There were 4349 patients, of which 4128 samples were successfully amplified (94.92%). Experts indicated that the failure of amplification might be attributed to low or absent numbers of bacteria and the presence of PCR inhibitors in the skin. During the treatment process with MDT, the resistance rate for RIF, DDS, and OFL combination was 10.18% (95% CI: 7.85 to 12.51), while the single resistance rates were 4.40% (95% CI: 3.02–5.77), 2.00% (95% CI: 2.48–5.06), and 1.73% (95% CI: 0.99–3.00), respectively. The results are similar to the previous studies [43,44]. For instance, in a large cohort study of leprosy established by several national sentinel testing centers, 8.0% of the strains had varying degrees of resistance (RIF: 3.8%, DDS: 4.5%, OFL: 1.10%, MDR: 1.24%).

We divided the study area into five parts based on the WHO criteria. It could be seen that the highest drug-resistance rate was in the Western Pacific region (17.05%, 95% CI: 7.17–26.93). Few papers from Europe and Africa were included, comprising 11.25% and 16.67%, respectively. Leprosy was almost extinct in Europe, and the first study on AMR in Europe was reported by Chauffour [35]: 18 cases were detected in 160 samples from France between 2001–2015. The results suggested that further studies on drug resistance were needed in Africa. The disease has placed a huge burden on the region, and incomplete MDT coverage or inadequate financial support may account for insufficient research. For example, Sofie MB recruited 1199 leprosy patients in Comoros (African region) between 2017–2019 and found that the patients were not resistant to any antimicrobial drugs [45]. Although the reasons for regional-level variation in resistance are beyond the scope of our study, the findings still reflect differences in antibiotics use (e.g., misuse) or availability. We can hypothesize that the distribution of resistant strains varies between regions, a finding that provides clues to explore the distribution or population mobility of resistant patients. In temporal subgroups, resistance rates were higher after 2009 than before (11.39% vs. 6.59%), which could be related to the global leprosy drug-resistance sentinel surveillance program established by WHO in 2009, resulting in more patients being detected [46]. We found a higher rate of drug resistance in relapsed patients than in new cases (14.26% vs. 7.25%), suggesting that patients were more likely to develop drug resistance if they received MDT for longer periods of time. In addition, we found that there are significant differences in the drug-resistance rates by sample size. Studies with <100 samples had significantly higher resistance rates than studies with ≥100 samples, indicating that sample size was associated with resistance rates.

DDS competes with *para*-aminobenzoate (PABA) and interrupts the function of the DHPS enzyme encoded by Folp1, disrupting folate biosynthesis [47,48]. In this study, the mutation rate of Folp1 was 4.40% (95% CI: 3.02–5.77). It is significantly lower than monotherapy for DDS [49]. In other words, leprosy monotherapy is more likely to induce mutations in drug-resistant genes. RIF is also one of the most effective and broad-spectrum antibiotics against leprosy, and the results indicated that the mutation rate of RpoB was 3.66% (95% CI: 2.41–4.90). The main mechanism of the drug acts via enzymatic activity inhibition of the β-subunit of RNAP holoenzyme, an enzyme determined by RpoB, which affects mRNA production and causes the death of Mycobacterium leprae [50]. As for the core drug in MDT, the emergence of RIF-resistant strains showed that the effectiveness of current leprosy control strategies is being challenged. DNA gyrase is an important enzyme in Mycobacterium that catalyzes ATP-dependent transient cleavage and negative supercoiling of closed-loop DNA [51]. GyrA determines the protein, and mutations within the gene are associated with resistance to ofloxacin. The mutation rate of GyrA was 1.28% (95% CI: 0.87~1.71). However, in a study conducted in Shandong Province, China, the mutation rate was 25.93%. The high resistance rate may be because the majority of patients in this region are from the rural population, and there is a phenomenon of antibiotic abuse, which leads to primary OFL-resistant strains [22].

Based on the 368 resistance strains, we analyzed the mutation rate of the gene codon. As the results indicated, Pro was the most common mutation locus of Folp1, with a rate of 83.97% (95% CI: 77.58–90.37), followed by the codon Thr (ES: 31.36%, 5.32~57.41). Sundeep also demonstrated that the mutations in target genes mostly involved these two amino acid loci [52]. We found that the pattern of Pro was replaced by Leu with a probability of 41.04% (95% CI: 22.76–59.31), followed by Arg (36.80%, 95% CI: 17.20~56.40). The highest mutation probability was found at the Ser on RpoB, 54.17% (53.02–69.40). It has been shown that the dynamics of protein phosphorylation at these two amino acid residues can regulate cellular activities in bacteria and eukaryotes [53]. In addition, the most common mutation pattern was Ser→Leu (42.95%, 95% CI: 22.65~63.25), which was also found in Mycobacterium tuberculosis, influenza virus, or *Escherichia coli* [54,55,56]. Indeed, the mutation feature of Ser→Leu has been demonstrated to significantly reduce the replication and hemagglutinin (HA) cleavage of the H1N2 swine influenza virus and is an influential factor in attenuating viral pathogenicity [57]. Therefore, we speculate that the viability and virulence of RIF-resistant strains will be reduced, but further validation is needed. The mutations in the GyrA target gene were mostly Ala, with a mutation rate of 75.18 % (95% CI: 68.54 to 88.62). Mutations in Ala can be considered a marker of resistance to OFL in Mycobacterium leprae. Furthermore, Ala→Val was the most common substitution pattern.

In summary, there are three distinctive features of drug resistance in leprosy. First, the global drug-resistance rate to MDT therapies is low, and the number of resistant strains decreases with fewer patients. Second, mutations in the resistance-determining regions of the target genes (Folp1, RpoB, and GyrA) were mainly involved in Pro, Ser, and Ala, and the resistant strains exhibit similar mutation patterns, such as Ser→Leu on RpoB. Similar results have also been found in other mycobacterial genera, such as Mycobacterium tuberculosis [57]. In the studies of resistant strains of *Mycobacterium tuberculosis*, mutations in different codons of RpoB may be associated with different levels of rifampicin resistance. For instance, mutations of Ser were associated with high levels of resistance to rifampicin (minimum inhibitory concentration [MIC] >64 μg/mL). It is well known that *Mycobacterium leprae* and *Mycobacterium tuberculosis* are highly homologous in the DRDR region of the RpoB gene. We can reasonably speculate that the viability and virulence of RIF-resistant strains may be increased. Experts also suggested that the RIF-resistant strains of *Mycobacterium leprae* have become better adapted to the drug and more virulent after RIF treatment [58,59,60]. Therefore, further validation is needed in future studies. At last, evidence of mutations in the resistance-determining region gene locus of *M. leprae* strains can help clinicians to select alternative treatment options for their patients, such as a combination of RIF, OFL, and clarithromycin. The emergence of resistance to OFL as a complementary therapy has also caused anxiety in patients. Patients who have never received quinolones for leprosy may have been treated with drugs for other infections, resulting in primary drug resistance [61]. The priority now is to establish a better resistance surveillance policy, careful post-treatment follow-up of cured patients, rapid identification of strains that may develop secondary resistance, earlier detection of recurrent cases and new treatment regimens, and more resistance investigations in endemic areas (e.g., Africa).

The present review has some limitations. Firstly, despite these subgroup analyses, there was still a high degree of heterogeneity in the included studies. This heterogeneity may reflect variability in sample collection methods or size. Secondly, the coverage of studies in some regions was low (e.g., Europe and Africa) and some articles contained a small sample size, which may have affected the final results. Thirdly, the meta-analysis only included drug-resistance mutation loci from these 25 articles, and there may be other mutations that have not yet been identified. At last, there were some potential publication biases in our meta-analysis, such as time lag bias or citation bias.

## Figures and Tables

**Table 1:** The study characteristics and quality assessment of 25 included papers in this systematic review.

Author	Publication Year	Study Period	Study Region	Total Positive Samples	Percentage * (%)	MDR	Folp1 (n/%)	RpoB (n/%)	GyrA (n/%)	JBI Scores
Charlotte et al. [19].	2016	2012–2015	Africa	24	100.00	0	3 (12.50)	1 (4.17)	0 (0.00)	6
Masanori et al. [20].	2011	2004–2009	Asia	290	68.56	0	19 (10.16)	7 (4.29)	0 (0.00)	6
Adalgiza et al. [21].	2012	2006–2008	South America	145	100.00	3	3 (5.26)	4 (7.02)	2 (3.51)	7
Liu et al. [22].	2015	2007–2014	Asia	85	100.00	3	1 (1.49)	5 (8.77)	21 (31.34)	6
Williams et al. [23].	2013	2011–2012	North America	39	100.00	0	2 (5.13)	1 (2.56)	0 (0.00)	6
Mallika, et al [24].	2014	2009–2013	Asia	111	79.29	2	9 (8.11)	4 (3.60)	9 (9.01)	6
Rosa et al. [25].	2020	2009–2013	South America	37	100.00	12	16 (59.26)	15 (60.00)	2 (7.41)	6
Lavania, et al. [26].	2015	2013–2014	Asia	215	93.02	0	1 (0.47)	7 (3.26)	0 (0.00)	7
Narang et al. [27].	2021	2019–2020	Asia	61	100.00	2	5 (8.20)	6 (9.84)	1 (1.64)	7
Niranjan et al. [28].	2020	2007–2018	Asia	77	92.77	2	2 (2.60)	1 (1.30)	5 (6.49)	6
Martha, et al [29].	2014	1985–2004	South America	941	100.00	6	4 (0.43)	27 (2.87)	10 (1.38)	6
Camilo et al. [30].	2016	2004–2013	South America	243	100.00	1	5 (2.06)	5 (2.06)	1 (0.41)	6
Lavania et al. [14].	2018	2009–2016	Asia	250	100.00	17	16 (6.40)	11 (4.40)	10 (4.00)	6
Chokkakula et al. [31].	2019	2013–2017	Asia	290	100.00	2	8 (2.76)	1 (0.34)	8 (2.76)	6
You et al. [32].	2005	NR	Asia	104	100.00	5	20 (19.23)	3 (2.88)	1 (0.96)	6
Chen et al. [33].	2019	2003–2011	Asia	76	100.00	1	19 (25.00)	0 (0.00)	1 (1.32)	7
Masanori et al. [34].	2010	NR	North America	38	100.00	0	0 (0.00)	2 (5.26)	1 (2.63)	6
Chauffour et al. [35].	2018	2001–2015	Europe	160	86.96	0	13 (8.13)	3 (1.88)	2 (1.25)	6
Singh et al. [36].	2018	NR	Asia	78	84.78	0	0 (0.00)	0 (0.00)	8 (32.00)	7
Masanori et al. [37].	2007	2000–2006	Asia	305	100.00	0	6 (4.58)	9 (11.54)	0 (0.00)	6
Matilde et al. [38].	2014	2009–2011	South America	197	100.00	1	0 (0.00)	4 (5.26)	0 (0.00)	5
Singh et al. [39].	2011	NR	South America	233	100.00	0	2 (0.86)	1 (0.43)	0 (0.00)	7
Abu et al. [40].	2015	2007–2009	Asia	50	100.00	NS	NS	2 (4.00)	NS	6
Sundeep et al. [41].	2014	NR	Asia	60	100.00	NS	NS	10 (16.67)	NS	6
Shi et al. [42].	2022	2018–2021	Asia	34	100.00		1 (3.13)	0 (0.00)	3.13	6

%: Positive Sample; NS: Not studied; MB: Multibacillary; PB: Paucibacillary. *: Percentage of positive samples out of all samples.

**Table 2:** Summarized estimates of drug resistance stratified by region (according to WHO criteria), different drugs, clinical treatment, relapse or new cases, and sample size variables.

Factor	Study/n	Cases/Positive Samples(n)	ES(%)/95% CI	I^2^	Heterogeneity Chi-Squared	*p*
WHO Region						
Southeast Asia	10	177/1482	11.43 (9.19–16.81)	88.8%	80.57	0.000
Americas	8	83/1873	4.19 (1.89–6.50)	82.6%	40.13	0.000
Western pacific	5	86/589	17.05 (7.17–26.93)	90.4%	41.60	0.974
Africa	1	4/24	16.67 (1.76–31.58)	-	-	-
Europe	1	18/160	11.25 (6.35–16.15)	-	-	-
Mediterranean						
Different drugs						
DDS	20	145/3325	3.98 (2.69–5.28)	86.2%	137.19	0.000
RIF	19	113/3352	2.97 (1.94–4.00)	70.6%	61.25	0.000
OFL	15	65/2609	1.90 (0.97–2.83)	72.6%	51.19	0.000
MDR	13	57/2617	1.73 (0.83–2.63)	71.7%	42.35	0.000
Relapsed or new cases						
New	14	128/1960	7.25 (4.65–9.84)	86.1%	93.74	0.000
Relapse	17	119/1248	14.26 (9.82–18.71)	82.9%	87.83	0.000
Clinical Treatment						
MB	15	175/2394	8.97 (6.29–11.65)	82.4%	79.57	0.000
PB	5	25/481	8.09 (2.15–14.02)	50.2%	8.03	0.091
No of isolation						
≥100	13	257/3469	7.69 (5.21–10.18)	91.5%	141.26	0.000
<100	13	111/659	15.00 (9.45–20.55)	80.4%	56.15	0.000
Study period						
After 2009	14	180/1652	11.39 (7.46–15.33)	91.6%	155.50	0.000
Before 2009	5	106/1785	6.59 (3.66–9.53)	82.3%	22.58	0.000
Overall	25	386/4128	10.18 (7.85–12.51)	89.7%	232.74	0.000

ES = drug-resistance rate, 95% CI = confidence interval.

**Table 3:** Mutation rate of drug-resistance genes.

Gene	Study/n	Mutations(n)	ES%	95% CI	I^2^ (%)	Heterogeneity Chi-Squared	*p*
**Folp1**	**20**	139	4.40	3.02-5.77	89.2	167.07	0.000
**RpoB**	**22**	129	3.66	2.41~4.90	80.2	105.84	0.000
**GyrA**	**16**	83	1.28	0.87~1.71	76.4	59.44	0.000

ES = drug-resistance rate, 95% CI = confidence interval.

**Table 4:** Genetic codon mutation characteristics.

Gene/Amino Acids	Study(n)	Events/ Mutations(n)	ES%	95% CI	I^2^ (%)	Heterogeneity Chi-Squared	*p*
**RpoB (codon: 439–459)**							
Ser (Serine)	10	58/73	54.17	53.02~69.40	79.10	43.06	0.000
Thr (Threonine)	3	5/24	19.54	3.88~35.20	0.00	0.62	0.733
Asp (Aspartic)	8	13/92	12.32	5.67~18.98	0.00	0.64	0.913
Gln (Glutamine)	6	12/51	19.29	8.88~29.71	0.00	4.14	0.529
Ala (Alanine)	3	6/21	24.52	6.98~42.06	5.20	2.11	0.348
Leu (Leucine)	3	3/25	7.47	−2.80~17.74	0.00	0.16	0.688
His (Histidine)	2	4/37	10.52	0.76~20.27	0.00	0.00	0.949
Val (Valine)	4	4/36	10.10	0.33~19.86	0.00	0.55	0.909
Gly (Glycine)	4	7/28	25.40	3.01~47.78	53.30	6.42	0.093
Phe (Phenylalanine)	2	2/18	10.13	−3.72~23.98	0.00	0.24	0.628
**Folp1 (codon: 44–64)**							
Pro (Proline)	10	87/112	83.97	77.58~90.37	37.80	14.47	0.107
Thr (Threonine)	11	44/133	31.36	5.32~57.41	95.60	229.51	0.000
Arg (Arginine)	2	2/37	5.31	−1.91~12.54	0.00	0.04	0.845
**GyrA (codon: 81–101)**							
Ala (Alanine)	5	31/68	75.18	68.54~88.62	63.60	11.00	0.027
Gly (Glycine)	3	7/24	26.00	10.14~41.88	40.80	3.38	0.185
Ser (Serine)	2	7/20	23.64	7.779~39.493	86.80	7.58	0.006

ES = drug-resistance rate, 95% CI = confidence interval.

## Data Availability

There are no relevant data for this manuscript other than those presented in the paper.

## Supplementary Materials

The supporting information can be downloaded at: https://www.mdpi.com/article/10.3390/ijms232012443/s1.
