# Supplementary material for: Drug Resistance (Dapsone, Rifampicin, Ofloxacin) and Resistance-Related Gene Mutation Features in Leprosy Patients: A Systematic Review and Meta-Analysis
Source: Int J Mol Sci. 2022 Oct 18;23(20):12443. doi: 10.3390/ijms232012443 (PMC9604410; doi:10.3390/ijms232012443)
Supplement: Supplementary file 1 [file ijms-23-12443-s001.zip › S1. Search stragegies.pdf]

**1. PUBMED （Human;English;166）**

**#1** (("Leprosy"[Mesh]) OR ((Hansen's Disease[Title/Abstract]) OR (Hansen Disease[Title/Abstract]))) OR ((((mycobacterium leprae[Title/Abstract]) OR (m leprae[Title/Abstract])) OR (m. leprae[Title/Abstract])) OR (mycobacterium lepromatosis[Title/Abstract]))

**#2** "resist"[All Fields] OR "resistance"[All Fields] OR "resistances"[All Fields] OR "resistant"[All Fields] OR "resistants"[All Fields] OR "resisted"[All Fields] OR "resistence"[All Fields] OR "resistences"[All Fields] OR "resistent"[All Fields] OR "resistibility"[All Fields] OR "resisting"[All Fields] OR "resistive"[All Fields] OR "resistively"[All Fields] OR "resistivities"[All Fields] OR "resistivity"[All Fields] OR "resists"[All Fields] OR "resist"[All Fields] OR "resistance"[All Fields] OR "resistances"[All Fields] OR "resistant"[All Fields] OR "resistants"[All Fields] OR "resisted"[All Fields] OR "resistence"[All Fields] OR "resistences"[All Fields] OR "resistent"[All Fields] OR "resistibility"[All Fields] OR "resisting"[All Fields] OR "resistive"[All Fields] OR "resistively"[All Fields] OR "resistivities"[All Fields] OR "resistivity"[All Fields] OR "resists"[All Fields].

**#3** (("genes"[MeSH Terms] OR "genes"[All Fields] OR "gene"[All Fields]) AND ("mutate"[All Fields] OR "mutated"[All Fields] OR "mutates"[All Fields] OR "mutating"[All Fields] OR "mutation"[MeSH Terms] OR "mutation"[All Fields] OR "mutations"[All Fields] OR "mutation s"[All Fields] OR "mutational"[All Fields] OR "mutator"[All Fields] OR "mutators"[All Fields])) OR ("drug"[All Fields] AND "resistance-conferring"[All Fields] AND ("mutate"[All Fields] OR "mutated"[All Fields] OR "mutates"[All Fields] OR "mutating"[All Fields] OR "mutation"[MeSH Terms] OR "mutation"[All Fields] OR "mutations"[All Fields] OR "mutation s"[All Fields] OR "mutational"[All Fields] OR "mutator"[All Fields] OR "mutators"[All Fields])) OR (("epidemiology"[MeSH Subheading] OR "epidemiology"[All Fields] OR "frequency"[All Fields] OR "epidemiology"[MeSH Terms] OR "frequence"[All Fields] OR "frequences"[All Fields] OR "frequencies"[All Fields]) AND ("genes"[MeSH Terms] OR "genes"[All Fields] OR "gene"[All Fields]) AND ("mutate"[All Fields] OR "mutated"[All Fields] OR "mutates"[All Fields] OR "mutating"[All Fields] OR "mutation"[MeSH Terms] OR "mutation"[All Fields] OR "mutations"[All Fields] OR "mutation s"[All Fields] OR "mutational"[All Fields] OR "mutator"[All Fields] OR "mutators"[All Fields])) OR (("magnitude"[All Fields] OR "magnitudes"[All Fields]) AND ("genes"[MeSH Terms] OR "genes"[All Fields] OR "gene"[All Fields]) AND ("mutate"[All Fields] OR "mutated"[All Fields] OR "mutates"[All Fields] OR "mutating"[All Fields] OR "mutation"[MeSH Terms] OR "mutation"[All Fields] OR "mutations"[All Fields] OR "mutation s"[All Fields] OR "mutational"[All Fields] OR "mutator"[All Fields] OR "mutators"[All Fields])) OR ("pathology, molecular"[MeSH Terms] OR ("pathology"[All Fields] AND "molecular"[All Fields]) OR "molecular pathology"[All Fields] OR ("molecular"[All Fields] AND "diagnostics"[All Fields]) OR "molecular diagnostics"[All Fields]) OR (("molecular"[All Fields] OR "moleculars"[All Fields]) AND ("detect"[All Fields] OR "detectabilities"[All Fields] OR "detectability"[All Fields] OR "detectable"[All Fields] OR "detectables"[All Fields] OR "detectably"[All Fields] OR "detected"[All Fields] OR "detectible"[All Fields] OR "detecting"[All Fields] OR "detection"[All Fields] OR "detections"[All Fields] OR "detects"[All Fields])) OR (("molecular"[All Fields] OR "moleculars"[All Fields]) AND ("characterisation"[All Fields] OR "characterisations"[All Fields] OR "characterise"[All Fields] OR "characterised"[All Fields] OR "characterises"[All Fields] OR "characterising"[All Fields] OR "characterization"[All Fields] OR "characterizations"[All Fields] OR "characterize"[All Fields] OR "characterized"[All Fields] OR "characterizes"[All Fields] OR "characterizing"[All Fields]))

**#1 AND #2 AND #3**

**2. Web of Science (n=424)**

**#1** ((((((TS=(leprosy)) OR TS=(Hansen's Disease)) OR TS=(Hansen Disease)) OR TS=(mycobacterium leprae)) OR TS=(m leprae)) OR TS=(m. leprae)) OR TS=(mycobacterium lepromatosis)
**#2** (TS=(resistance)) OR TS=(resistant)
**#3** ((((((TS=(gene mutations)) OR TS=(drug resistance-conferring mutations)) OR TS=(frequency of gene mutations)) OR TS=(magnitudes of gene mutations)) OR TS=(molecular diagnostics)) OR TS=(molecular detection)) OR TS=(molecular characterization)

**#1 AND #2 AND #3**

**3. Scoups（n=465）**

**#1** ( TITLE-ABS-KEY ( "leprosy" ) OR TITLE-ABS-KEY ( "hansens disease" ) OR TITLE-ABS-KEY ( "hansen disease" ) OR TITLE-ABS-KEY ( "mycobacterium leprae" ) OR TITLE-ABS-KEY ( "m leprae" ) OR TITLE-ABS-KEY ( "m. leprae" ) OR TITLE-ABS-KEY ( "mycobacterium lepromatosis" ) )
**#2** ( ALL ( "gene mutations" ) OR ALL ( "drug resistance-conferring mutations" ) OR ALL ( "frequency of gene mutations" ) OR ALL ( "magnitudes of gene mutations" ) OR ALL ( "molecular diagnostics" ) OR ALL ( "molecular detection" ) OR ALL ( "molecular characterization" ) )
**#3** ( ALL ( resistance ) OR ALL ( resistant ) )
**#1 AND #2 AND #3**

**4. Embase (n=42)**

**#1** 'leprosy' OR 'hansens disease' OR 'hansen disease' OR 'mycobacterium leprae' OR 'm leprae' OR 'm. leprae' OR 'mycobacterium lepromatosis'

**#2** resistance OR resistant

**#3** 'gene mutations' OR 'drug resistance-conferring mutations' OR 'frequency of gene mutations' OR 'magnitudes of gene mutations' OR 'molecular diagnostics' OR 'molecular detection' OR 'molecular characterization'

**#1 AND #2 AND #3**
