# Supplementary material for: Drug Resistance (Dapsone, Rifampicin, Ofloxacin) and Resistance-Related Gene Mutation Features in Leprosy Patients: A Systematic Review and Meta-Analysis
Source: Int J Mol Sci. 2022 Oct 18;23(20):12443. doi: 10.3390/ijms232012443 (PMC9604410; doi:10.3390/ijms232012443)
Supplement: Supplementary file 1 [file ijms-23-12443-s001.zip › S2. JBI Scoring Results.pdf]

Additnal file: Table S1. Quality appraisal result of included studies; Using Joanna Briggs Institute (JBI) quality appraisal checklist for cross-sectional studies.

Authors, and Year	Q1	Q2	Q3	Q4	Q5	Q6	Q7	Q8	Scores (0~8)	Publication
Charlotte et al. [1].	Yes	Yes	Yes	Yes	Yes	No	Yes	No	6	Clinical infectious diseases
Masanori et al. [2].	Yes	No	Yes	Yes	Yes	Yes	Yes	No	6	Clinical infectious diseases
Adalgiza et al. [3].	Yes	No	Yes	Yes	Yes	Yes	Yes	Yes	7	Journal of clinical microbiology
Liu et al. [4]	Yes	Yes	Yes	Yes	No	Yes	Yes	No	6	Clinical and experimental dermatology
Williams et al. [5].	Yes	Yes	Yes	Yes	Yes	No	Yes	No	6	Clinical Infectious Diseases
Mallika et al [6].	Yes	Yes	Yes	Yes	Yes	No	Yes	No	6	Leprosy review
Rosa et al. [7].	Yes	Yes	Yes	No	Yes	Yes	Yes	No	6	Clinical infectious diseases
Lavania et al. [8].	Yes	Yes	Yes	Yes	Yes	Yes	Yes	No	7	Clinical microbiology and infection
Narang et al. [9].	Yes	Yes	Yes	Yes	Yes	No	Yes	Yes	7	Clinical and experimental dermatology
Niranjan et al. [10].	Yes	Yes	Yes	Yes	No	No	Yes	Yes	6	The American journal of tropical medicine and hygiene
Martha et al [11].	Yes	No	Yes	Yes	Yes	No	Yes	Yes	6	Biomedica
Camilo et al. [12].	Yes	Yes	Yes	Yes	No	No	Yes	Yes	6	PLoS neglected tropical diseases
Lavania et al. [13].	Yes	Yes	Yes	Yes	No	No	Yes	Yes	6	Journal of global antimicrobial resistance
Chokkakula et al. [14].	Yes	Yes	Yes	Yes	No	No	Yes	Yes	6	Emerging microbes & infections
You et al. [15].	Yes	Yes	Yes	Yes	Yes	No	Yes	No	6	The Journal of infection
Chen et al. [16].	Yes	Yes	Yes	No	Yes	Yes	Yes	Yes	7	PLoS neglected tropical diseases

Masanori et al. [17].	Yes	No	Yes	Yes	Yes	No	Yes	Yes	6	Japanese journal of infectious diseases
Chauffour et al. [18].	Yes	Yes	Yes	Yes	Yes	No	Yes	No	6	Clinical microbiology and infection
Singh et al. [19].	Yes	Yes	Yes	Yes	Yes	No	Yes	Yes	7	Indian journal of dermatology, venereology and leprology
Masanori et al. [20].	Yes	No	Yes	Yes	Yes	No	Yes	Yes	6	Leprosy review
Matilde et al. [21].	Yes	No	Yes	Yes	Yes	No	Yes	No	5	Journal of clinical microbiology
Singh et al. [22].	Yes	Yes	Yes	Yes	Yes	No	Yes	Yes	7	Antimicrobial agents and chemotherapy
Abu et al. [23].	Yes	Yes	Yes	Yes	Yes	No	Yes	No	6	Indian journal of dermatology, venereology and leprology
Sundeep et al. [24].	Yes	Yes	Yes	Yes	Yes	No	Yes	No	6	Medical microbiology and immunology
Shi et al. [25].	Yes	Yes	Yes	Yes	No	No	Yes	Yes	6	Infection Drug Resistance

Q1: Were the criteria for inclusion in the sample clearly defined?
Q2: Were the study subjects and the setting described in detail?
Q3: Was the exposure measured in a valid and reliable way?
Q4: Were objective, standard criteria used for measurement of the condition?
Q5: Were confounding factors identified?
Q6: Were strategies to deal with confounding factors stated?
Q7: Were the outcomes measured in a valid and reliable way?
Q8: Was appropriate statistical analysis used?

1. Avanzi, C.; Busso, P.; Benjak, A.; Loiseau, C.; Fomba, A.; Doumbia, G.; Camara, I.; Lamou, A.; Sock, G.; Drame, T.; et al. Transmission of Drug-Resistant Leprosy in Guinea-Conakry Detected Using Molecular Epidemiological Approaches: Table 1. *Clin. Infect. Dis.* **2016**, *63*, 1482–1484. https://doi.org/10.1093/cid/ciw572.
2. Kai, M.; Nguyen Phuc, N.H.; Nguyen, H.A.; Pham T.; Nguyen, K.H.; Miyamoto, Y.; Maeda, Y.; Fukutomi, Y.; Nakata, N.; Matsuoka, M.; et al. Analysis of drug-resistant strains of *Mycobacterium leprae* in an endemic area of Vietnam. *Clin. Infect. Dis.* **2011**, *52*, e127–e132.
3. Rocha, A.D.S.; Cunha, M.D.G.; Diniz, L.M.; Salgado, C.; Aires, M.A.P.; Nery, J.A.; Gallo, E.N.; Miranda, A.; Magnanini, M.M.F.; Matsuoka, M.; et al. Drug and Multidrug Resistance among *Mycobacterium leprae* Isolates from Brazilian Relapsed Leprosy Patients. *J. Clin. Microbiol.* **2012**, *50*, 1912–1917. https://doi.org/10.1128/JCM.06561-11.
4. Liu, D.; Zhang, Q.; Sun, Y.; Wang, C.; Zhang, Y.; Fu, X.; Chen, M.; Zhou, G.; Yu, X.; Wang, J.; et al. Drug resistance in *Mycobacterium leprae* from patients with leprosy in China. *Clin. Exp. Dermatol.* **2015**, *40*, 908–911.
5. Williams, D.L.; Lewis, C.; Sandoval, F.G.; Robbins, N.; Keas, S.; Gillis, T.P.; Scollard, D.M. Drug resistance in patients with leprosy in the United States. *Clin. Infect. Dis.* **2014**, *58*, 72–73.
6. Lavania, M.; Jadhav, R.S.; Chaitanya, V.S.; Turankar, R.P.; Selvasekhar, A.; Das, L.; Darlong, F.; Hambroom, U.K.; Kumar, S.; Sengupta, U. Drug resistance patterns in *Mycobacterium leprae* isolates from relapsed leprosy patients attending. The Leprosy Mission (TLM) Hospitals in India. *Lepr. Rev.* **2014**, *85*, 177–185.
7. Rosa, P.S.; D'Espindula, H.R.S.; Melo, A.C.L.; Fontes, A.N.B.; Finardi, A.J.; Belone, A.F.F.; Sartori, B.G.C.; Pires, C.A.A.; Soares, C.T.; Marques, F.B.; et al. Emergence and Transmission of Drug-/Multidrug-resistant *Mycobacterium leprae* in a Former Leprosy Colony in the Brazilian Amazon. *Clin. Infect. Dis.* **2020**, *70*, 2054–

2061.
8. Lavania, M.; Nigam, A.; Turankar, R.; Singh, I.; Gupta, P.; Kumar, S.; Sengupta, U.; John, A. Emergence of primary drug resistance to rifampicin in *Mycobacterium leprae* strains from leprosy patients in India. *Clin. Microbiol. Infect.* **2015**, *21*, e85–e86.
9. Narang, T.; Kamat, D.; Thakur, V.; Lavania, M.; Singh, I.; Ahuja, M.; Dogra, S. Equal rates of drug resistance in leprosy cases with relapse and recurrent/chronic Type 2 reaction: Time to revise the guidelines for drug-resistance testing in leprosy? *Clin. Exp. Dermatol.* **2022**, *47*, 297–302.
10. Mahajan, N.P.; Lavania, M.; Singh, I.; Nashi, S.; Preethish-Kumar, V.; Vengalil, S.; Polavarapu, K.; Pradeep-Chandra-Reddy, C.; KeerthiPriya, M.; Mahadevan, A.; et al. Evidence for *Mycobacterium leprae* Drug Resistance in a Large Cohort of Leprous Neuropathy Patients from India. *Am. J. Trop. Med. Hyg.* **2020**, *3*, 547–552.
11. Guerrero, M.I.; Colorado, C.L.; Torres, J.F.; León, C.I. Is drug-resistant *Mycobacterium leprae* a real cause for concern? First approach to molecular monitoring of multibacillary Colombian patients with and without previous leprosy treatment. *Biomed. Rev. Del Inst. Nac. Salud* **2014**, *34* (Suppl. 1), 137–147.
12. Beltrán-Alzate, C.; Díaz, F.L.; Romero-Montoya, M.; Sakamuri, R.; Li, W.; Kimura, M.; Brennan, P.; Cardona-Castro, N. Leprosy Drug Resistance Surveillance in Colombia: The Experience of a Sentinel Country. *PLoS Negl. Trop. Dis.* **2016**, *10*, e0005041.
13. Lavania, M.; Singh, I.; Turankar, R.P.; Ahuja, M.; Pathak, V.; Sengupta, U.; Das, L.; Kumar, A.; Darlong, J.; Nathan, R.; et al. Molecular detection of multidrug-resistant *Mycobacterium leprae* from Indian leprosy patients. *J. Glob. Antimicrob. Resist.* **2018**, *12*, 214–219.
14. Chokkakula, S.; Chen, Z.; Wang, L.; Jiang, H.; Chen, Y.; Shi, Y.; Zhang, W.; Gao, W.; Yang, J.; Li, J.; et al. Molecular surveillance of antimicrobial resistance and transmission pattern of *Mycobacterium leprae* in Chinese leprosy patients. *Emerg. Microbes Infect.* **2019**, *8*, 1479–1489. https://doi.org/10.1080/22221751.2019.1677177.
15. You, E.Y.; Kang, T.J.; Kim, S.K.; Lee, S.B.; Chae, G.T. Mutations in genes related to drug resistance in *Mycobacterium leprae* isolates from leprosy patients in Korea. *J. Infect.* **2005**, *50*, 6–11.
16. Chen, X.; He, J.; Liu, J.; You, Y.; Yuan, L.; Wen, Y. Nested PCR and the TaqMan SNP Genotyping Assay enhanced the sensitivity of drug resistance testing of *Mycobacterium leprae* using clinical specimens of leprosy patients. *PLoS Negl. Trop. Dis.* **2019**, *13*, e0007946.
17. Matsuoka, M.; Suzuki, Y.; Garcia, I.E.; Fafutis-Morris, M.; Vargas-González, A.; Carreño-Martinez, C.; Fukushima, Y.; Nakajima, C. Possible mode of emergence for drug-resistant leprosy is revealed by an analysis of samples from Mexico. *Jpn. J. Infect. Dis.* **2010**, *63*, 412–416.
18. Chauffour, A.; Lecorche, E.; Reibel, F.; Mougari, F.; Raskine, L.; Aubry, A.; Jarlier, V.; Cambau, E. Prospective study on antimicrobial resistance in leprosy cases diagnosed in France from 2001 to 2015. *Clin. Microbiol. Infect.* **2018**, *24*, 1213.e5–1213.e8.
19. Singh, S.; Kumar, A.; Nath, G.; Singh, T.; Mishra, M. Resistance to anti leprosy drugs in multi-bacillary leprosy: A cross sectional study from a tertiary care centre in eastern Uttar Pradesh, India. *Indian J. Dermatol. Venereol. Leprol.* **2018**, *84*, 275–279.
20. Matsuoka, M.; Budiawan, T.; Aye, K.S.; Kyaw, K.; Tan, E.V.; Cruz, E.D.; Gelber, R.; Saunderson, P.; Balagon, V.; Pannikar, V. The frequency of drug resistance mutations in *Mycobacterium leprae* isolates in untreated and relapsed leprosy patients from Myanmar, Indonesia and the Philippines. *Lepr. Rev.* **2007**, *78*, 343–352.
21. Mejía, M.D.C.C.; Dos Santos, M.P.; da Silva, G.A.V.; Passos, I.D.M.; Naveca, F.G.; Cunha, M.D.G.S.; Moraes, M.O.; de Paula, L. Identification of primary drug resistance to rifampin in *Mycobacterium leprae* strains from leprosy patients in Amazonas State, Brazil. *J. Clin. Microbiol.* **2014**, *52*, 4359–4360.
22. Singh, P.; Busso, P.; Paniz-Mondolfi, A.; Aranzazu, N.; Monot, M.; Honore, N.; Belone, A.D.F.F.; Virmond, M.; Villarreal-Olaya, M.E.; Rivas, C.; et al. Molecular Drug Susceptibility Testing and Genotyping of *Mycobacterium leprae* Strains from South America. *Antimicrob. Agents Chemother.* **2011**, *55*, 2971–2973.
23. Bhattacharya, B.; Reja, A.H.; Biswas, N.; Biswas, S.; Lavania, M.; Chaitanya, V.; Banerjee, S.; Patra, P.M.; Gupta, U.; Patra, P.; et al. Report of rpoB mutation in clinically suspected cases of drug resistant leprosy: A study from Eastern India. *Indian J. Dermatol. Venereol. Leprol.* **2015**, *81*, 155–161.
24. Vedithi, S.C.; Lavania, M.; Kumar, M.; Kaur, P.; Turankar, R.P.; Singh, I.; Nigam, A.; Sengupta, U. A report of rifampin-resistant leprosy from northern and eastern India: Identification and in silico analysis of molecular interactions. *Med. Microbiol. Immunol.* **2015**, *204*, 193–203. https://doi.org/10.1007/s00430-014-0354-1.
25. Shi, Y.; Kong, W.; Jiang, H.; Zhang, W.; Wang, C.; Wu, L.; Shen, Y.; Yao, Q.; Wang, H. Molecular Surveillance of Antimicrobial Resistance of *Mycobacterium leprae* from Leprosy Patients in Zhejiang Province, China. *Infect. Drug Resist.* **2022**, *15*, 4029–4036. https://doi.org/10.2147/IDR.S368682.
